# A *Lactobacillus* Combination Ameliorates Lung Inflammation in an Elastase/LPS—induced Mouse Model of Chronic Obstructive Pulmonary Disease

**DOI:** 10.1007/s12602-024-10300-9

**Published:** 2024-06-12

**Authors:** Huan-Ting Shen, Yi-Ting Fang, Wan-Hua Tsai, Chia-Hsuan Chou, Ming-Shyan Huang, Yao-Tsung Yeh, Jiun-Ting Wu, Cheng-Hsieh Huang, Bing-Yen Wang, Wen-Wei Chang

**Affiliations:** 1 Department of Pulmonary Medicine, Taichung Tzu Chi Hospital, Buddhist Tzu Chi Medical Foundation, No. 88, Sec. 1, Fengxing Rd., Tanzi Dist., Taichung City, 427003 Taiwan; 2 Research and Development Department, GenMont Biotech Incorporation, No.8, Nanke 7th Rd., Shanhua Dist., Tainan City, 741014 Taiwan; 3 Division of Respiratory and Chest Medicine, Department of Internal Medicine, E-Da Cancer Hospital, No. 1, Yida Rd, Yanchao Dist, Kaohsiung City, 824005 Taiwan; 4 Aging and Disease Prevention Research Center, Fooyin University, No. 151, Jinxue Rd., Daliao Dist., Kaohsiung City, 831301 Taiwan; 5 Division of Thoracic Surgery, Department of Surgery, Changhua Christian Hospital, No. 135, Nanhsiao Street, Changhua County 500209 Taiwan; 6 Department of Post-Baccalaureate Medicine, College of Medicine, National Chung Hsing University, No. 145, Xingda Rd., South Dist., Taichung City, 402202 Taiwan; 7 Department of Biomedical Sciences, Chung Shan Medical University, No.110, Sec.1, Jianguo N.Rd, Taichung City, 402306 Taiwan; 8 Department of Medical Research, Chung Shan Medical University Hospital, No.110, Sec.1, Jianguo N.Rd, Taichung City, 402306 Taiwan

**Keywords:** COPD, *Lactobacillus*, Gut microbiota, Gut-lung axis, Short chain fatty acids

## Abstract

Chronic obstructive pulmonary disease (COPD) is the world's leading lung disease and lacks effective and specific clinical strategies. Probiotics are increasingly used to support the improvement of the course of inflammatory diseases. In this study, we evaluated the potential of a lactic acid bacteria (LAB) combination containing *Limosilactobacillus reuteri* GMNL-89 and *Lacticaseibacillus paracasei* GMNL-133 to decrease lung inflammation and emphysema in a COPD mouse model. This model was induced by intranasal stimulation with elastase and LPS for 4 weeks, followed by 2 weeks of oral LAB administration. The results showed that the LAB combination decreased lung emphysema and reduced inflammatory cytokines (IL-1β, IL-6, TNF-α) in the lung tissue of COPD mice. Microbiome analysis revealed that *Bifidobacterium* and *Akkermansia muciniphila*, reduced in the gut of COPD mice, could be restored after LAB treatment. Microbial α-diversity in the lungs decreased in COPD mice but was reversed after LAB administration, which also increased the relative abundance of *Candidatus arthromitus* in the gut and decreased *Burkholderia* in the lungs. Furthermore, LAB-treated COPD mice exhibited increased levels of short-chain fatty acids, specifically acetic acid and propionic acid, in the cecum. Additionally, pulmonary emphysema and inflammation negatively correlated with *C. arthromitus* and *Adlercreutzia* levels. In conclusion, the combination of *L. reuteri* GMNL-89 and *L. paracasei* GMNL-133 demonstrates beneficial effects on pulmonary emphysema and inflammation in experimental COPD mice, correlating with changes in gut and lung microbiota, and providing a potential strategy for future adjuvant therapy.

**Supplementary Information:**

The online version contains supplementary material available at 10.1007/s12602-024-10300-9.

## Introduction

Chronic obstructive pulmonary disease (COPD) is characterized by irreversible obstruction of the airways and is associated with an inflammatory disorder of the airways [1, 2]. Several etiological factors, such as cigarette smoking, long-term exposure to air pollution, and pathogenic infections, pose a high risk for the development of COPD [3–5]. Lung parenchymal destruction (emphysema), central airway inflammation (chronic bronchitis), and peripheral airway inflammation (respiratory bronchiolitis) are the pathological hallmarks of COPD [6]. The chronic inflammation of COPD is considered to result from immune cell infiltration and accumulation at the local lung site, with high levels of inflammatory cytokines, including IL-1β, IL-6, TNF-α, and chemokines/chemokine receptors, including CXC chemokine ligand 1 (CXCL1), CXCL8, CXC chemokine receptor 3, triggering lymphoid aggregates, magnifying downstream inflammatory effects, and increasing cytotoxic activity [7–9]. Although several targeted drugs for COPD aim to block lung inflammation—such as infliximab, which blocks TNF-α—current pharmacological therapies still have limited effects and show few valuable results in clinical trials [10, 11].

Changing the gut microbiota through probiotic intervention is closely linked to improvements lung inflammation and injury [12]. Several metabolites or products, including short-chain fatty acids (SCFAs) and amino acids, from the gut microbiota also contribute to respiratory health through the gut-lung axis [13, 14]. Probiotics such as *Lactobacillus* and *Bifidobacterium* show anti-inflammatory effects by decreasing pulmonary inflammation in COPD mouse models [15, 16]. In our previous study, *L. reuteri* GMNL-89 was shown to have a beneficial effect in the attenuation of systemic lupus erythematosus-induced hepatitis and liver injury [17]. Moreover, the inhibitory effect of gemcitabine on tumor growth in pancreatic cancer can be synergistically enhanced by the intervention of probiotic compositions, *L. reuteri* GMNL-89 and *L. paracasei* GMNL-133 [18].

Despite the limited effectiveness of current pharmacological therapy and the largely unknown pathogenesis of COPD, it is worthwhile to clarify the relationship between gut and lung microbiota with lung inflammation in COPD. Previously, we found that *L. reuteri* GMNL-89 has an anti-inflammatory effect on liver inflammation in lupus-prone mice [17], and *L. paracasei* GMNL-133 reduces symptoms in children with atopic dermatitis [19]. Additionally, several studies suggest that probiotic combinations may be more effective than single strains [19–23]. In this study, we use a combination of GMNL-89 and GMNL-133 in a mouse model of COPD to investigate whether a probiotic intervention can ameliorate conditions such as emphysema and lung inflammation, by reducing immune cell infiltration and pro-inflammatory cytokine levels. In addition, we aim to elucidate the metabolites of the gut microbiota that influence the disease process of COPD through the gut-lung axis. Correlation between lung inflammation and gut/lung microbiota in COPD mouse will be observed.

## Materials and Methods

### Animals and Treatment

**Fig. 1:**
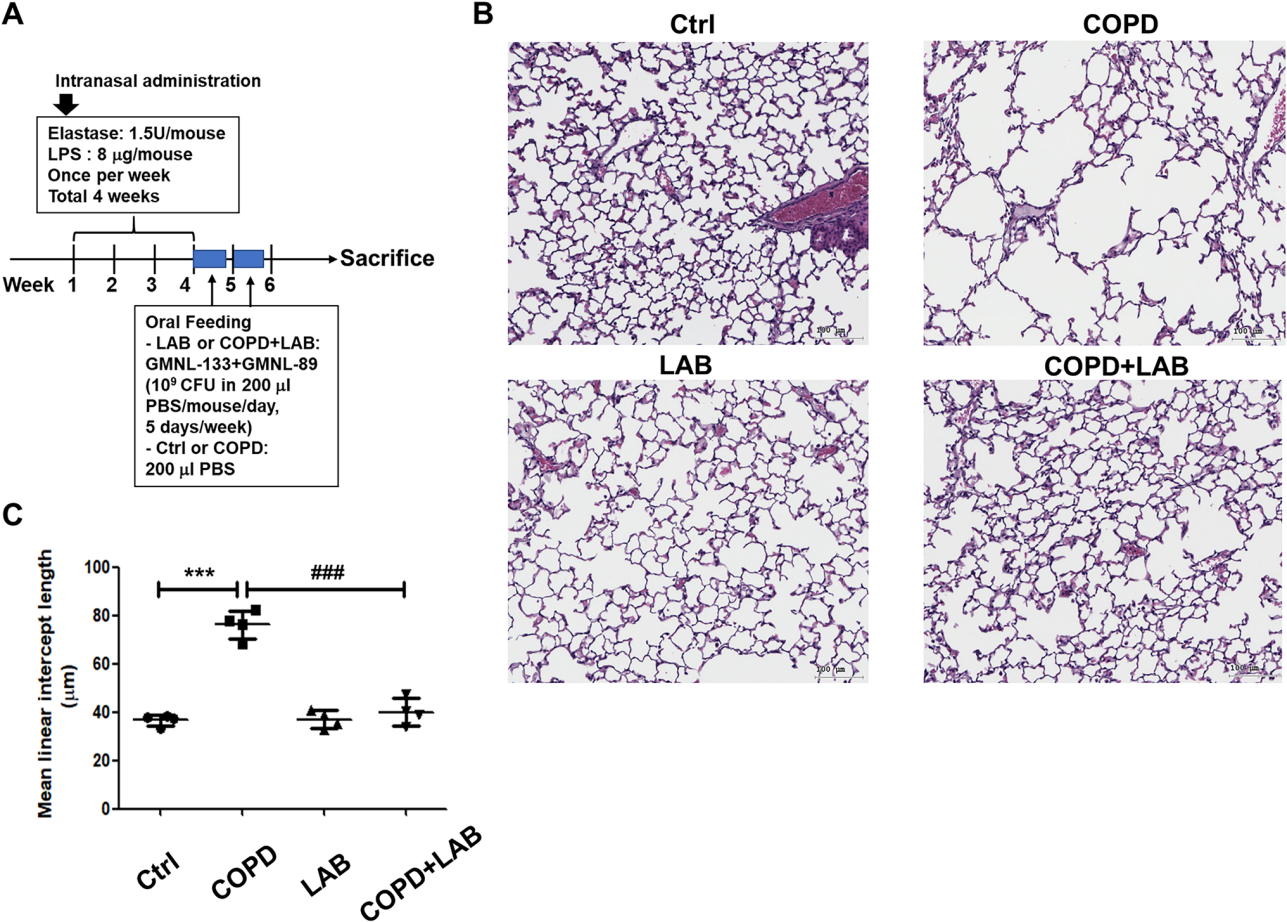
Oral administration of LAB containing GMNL-89 and GMNL-133 ameliorates pulmonary emphysema in LPS/elastase-induced COPD mice. (**A**) The timeline for inducing COPD and inoculating mice (n = 4 per group) with LAB, consisting of GMNL-89 and GMNL-133, was illustrated according to the descriptions in the Methods section. (**B**) Histological analysis of lung tissues was performed by paraffin sectioning and H&E staining. (**C**) The mean linear intercept of the lung tissues was used to evaluate the degree of lung emphysema, which was determined using ImageJ software. Ctrl, naïve mice used as control; COPD, intranasal treatment with elastase and LPS; LAB, administration of LAB without induction of COPD; COPD + LAB, induction of COPD followed by oral administration of LAB. *** p < 0.001, compared with Ctrl group. ### p < 0.001, compared with COPD

BALB/cByJNarl male mice aged 8–10 weeks, were obtained from the National Laboratory Animal Centre, Tainan, Taiwan. The study protocol was approved by the IACUC Laboratory Animal Center of GenMont Biotech Incorporation (Taiwan IACUC No. 194, GenMont Biotech Incorporation No. 110009). The mouse model of COPD was established by exposure to elastase and LPS, as described in a previous study by Ganesan et al. [24]. Briefly, COPD was induced via the intranasal route with 1.5 U of porcine pancreatic elastase (Cat No. E7885, Sigma-Aldrich, St. Louis, MO, USA) on day 1 and 8 µg of LPS from *E. coli* O26:B6 (Cat. No. L2654, Sigma-Aldrich) on day 4 for four consecutive weeks. Mice that received a phosphate-buffered saline (PBS) solution served as the control group. The experimental procedure is shown in Fig. 1A. After exposure to elastase/LPS for four weeks, each mouse in the LAB or COPD/LAB groups was orally gavaged with 10^9^ colony-forming unit (CFU) of probiotic compositions (GMNL-89 plus GMNL-133 at a ratio of 1:1) in 200 µl of PBS every day, five days per week for two consecutive weeks. The control mice were fed 200 µl of PBS by oral gavage. After two weeks of probiotic administration, the mice were sacrificed.

### Bronchoalveolar Lavage Fluid (BALF) Collection and Lung Histology Analysis

The collection of BALF was conducted according to the protocol described by Van Hoecke et al. [25]. The fluids were then centrifuged at 1500 g for 10 min to separate the cell pellet and the supernatant. Total cell counts of the BALFs were measured under light microscopy after staining with Giemsa. The cells were collected by centrifugation, resuspended in PBS, and analyzed by flow cytometry for infiltrated immune cells, which were stained with fluorescence-conjugated antibodies including anti-CD45, anti-CD11b, and anti-Ly6G. Macrophages and neutrophils were defined as CD45^+^/CD11b^+^ [26] and CD45^+^/Ly6G^+^ [27], respectively, and quantified by calculating the total number of BALF cells multiplied by the percentage of each cell type. For histological analysis, the left lung tissues from the sacrificed mice were perfused transtracheally with 10% formalin in PBS. The samples were embedded in paraffin, sectioned at 5 µm thickness, and stained with hematoxylin and eosin. The mean linear intercept length (MLI) was used to assess emphysematous changes in the lungs using ImageJ software (version 1.53v, National Institutes of Health, Bethesda, MD, USA), based on the analytical settings by Crowley et al. [28]. The levels of IL-6 and TNF-α were determined by immunohistochemistry using the biotin-avidin method. Images of the slides were captured and analyzed using the TissueFAX Plus Imaging System (TissueGnostics, Vienna, Austria).

### DNA Extraction and Analysis of Microbiota By Quantitative PCR (qPCR)

DNA from the fecal samples was extracted using the Quick-DNA fungal/bacterial kit (Zymo Research Corporation, Irvine, CA, USA). Reaction mixtures were prepared with 5 µl of 2 × Rotor-Gene SYBR Green PCR Master Mixes (QIAGEN, Hilden, Germany), 2 µl of DNA from fecal extract and 3 µl of forward and reverse primers. The primer sequences of the targeted microbes (*L. reuteri*, *L. paracasei*, *Lactobacillus* spp., *Bifidobacterium*, *Akkermansia muciniphila*) and the internal control (total bacteria) are listed in Table 1. The reaction mixtures were analyzed on the Roter Gene Q 2plex machine (QIAGEN). Relative quantification of changes in specific bacterial species was calculated using the Eq. 2^−△△Ct^. ΔCt was calculated as the difference between the Ct of the target bacteria primers and the Ct of the total bacteria primers (Ct_target bacteria − Ct_total bacteria). ΔΔCt was calculated as the difference between the ΔCt measured in each treatment and the ΔCt measured in the control group. Values derived from the 2^−ΔΔCt^ method represent fold changes between a treated sample and a control.

**Table 1:** The primer sequences of selected bacteria

Name	Forward primer (5’ to 3’)	Reverse primer (5’ to 3’)
Total bacteria	GTGSTGCAYGGYTGTCGTCA	ACGTCRTCCMCACCTTCCTC
*L. reuteri*	GCGTTGATGTTGTTGAAGGAATGAGCTTTG	CATCAGCAATGATTAAGAGAGCACGGCC
*L. paracasei*	CAGACACAGATCAGCTTG	AACTTGGCATCCTTCAAA
*Lactobacillus* spp.	TGGAAACAGRTGCTAATACCG	GTCCATTGTGGAAGATTCCC
*Bifidobacterium*	CGCGTCYGGTGTGAAAG	CCCCACATCCAGCATCCA
*A. muciniphila*	CAGCACGTGAAGGTGGGGAC	CCTTGCGGTTGGCTTCAGAT

### 16S Ribosomal RNA Gene Amplicon Sequencing

Inferior lobe of the lung and ileum intestine samples from each group were collected at the sacrifice timepoint, as shown in Fig. 1A, and all specimens were extracted using a QIAGEN DNA kit according to the manufacturer’s instructions. DNA samples were analyzed with a 260/280 OD in the range of 1.8 to 2.0. For 16S ribosomal RNA (rRNA) amplicons, PCR was performed by using metagenomic DNA as a template, which was amplified with the bacterial-specific primers S17 (5’-TCG TCG GCA GCG TCA GAT GTG TAT AAG AGA CAG CCT ACG GGN GGC WGC AG-3’) and A21 (5’-GTC TCG TGG GCT CGG AGA TGT GTA TAA GAG ACA GGA CTA CHV GGG TAT CTA ATC C-3’). A fragment analyzer (5300; Agilent Technologies) was used to verify the amplified DNA sizing, and an Illumina MiSeq platform was used to sequencing. DNA samples were assigned indices and Illumina sequencing adapters with the Nextera XT Index Kit v2. After library construction, the samples were mixed using a 600-cycle MiSeq Reagent Kit v3 at a final concentration of 4 pM, loaded onto a MiSeq cartridge, and then transferred onto the instrument. Automated cluster generation and a 2 × 300 bp paired-end sequencing run were performed. The sequences generated passed through a filtering process to obtain the qualified reads. The total reads were merged, low-quality and chimera sequences were removed, and OTUs at a 97% similarity with the Greengenes database (v13.8) were clustered. We employed the QIAGEN CLC Microbial Genomics Module (v10.1.1) for further analysis.

### Bioinformatic Analysis

The processing and statistical analysis of meta-taxonomic data were performed as previously described [29]. To determine differences in microbial composition among groups, the Shannon diversity index was used to analyze the alpha diversity of the taxonomic composition. The principal coordinate analysis (PCoA) with weighted UniFrac was used to measure the beta diversity. The Qiagen CLC Microbial Genomics Module was used in combination with linear discriminant analysis effect size (LEfSe) to produce the OTU table. To identify specific microbial markers among groups, LEfSe was performed using the Galaxy/Hutlab web tool with a 0.05 alpha value as the cutoff for the factorial Kruskal–Wallis test, pairwise Wilcoxon test, and a linear discriminant analysis (LDA) score cutoff of 2.0. Spearman’s correlation (calculated using the corrplot package v0.84) and principal component analysis (PCA; performed using the ade4 package v1.7–16) were applied using the R language (v4.0.2).

### Analysis and Measurement of SCFAs

Gas chromatography with flame ionization detection (GC-FID) was used to measure the concentrations of five SCFAs: acetic acid (AA), propionic acid (PA), butyric acid (BA), iso-BA, and pentanoic acid (PenA) in cecal contents [30], as performed by the Health Technology Center in Chung Shan Medical University (Taichung, Taiwan). Briefly, 5 µL of the 100 µM internal standard was mixed with 100 µL of the sample and 100 µL of propyl formate. After vortexing and centrifugation, the supernatant was subjected to GC analysis using an Agilent 7890A gas chromatograph. A sample of 0.2–0.4 g of cecal matter was dissolved in reverse osmosis water and vortexed for 2 min. The mixture was then centrifuged at 6000 × g for 5 min. The suspension was collected and filtered through a 0.45 µm filter. 1 mL of the filtered sample was acidified with 2.0 mL of 50% sulfuric acid, extracted with 1 mL of ether using a vortexing device for 2 min, and then centrifuged at 4000 × g for 5 min. After resting at 4 °C for 30 min, the ether fraction of the suspension was used for GC-FID analysis. Each SCFA was quantified in units of µmole/g of cecal content.

### Statistical Analysis

Experimental results were expressed as mean ± standard deviation (SD) and expressed as percentage of treatment groups in comparison with control groups. For normally distributed data, one-way ANOVA with post hoc Tukey's multiple comparison test was used for more than two groups, and Student's *t*-test was used for two groups. A P value of less than 0.05 was considered statistically significant.

## Results

### *L. reuteri *GMNL-89 and *L. paracasei* GMNL-133 Oral Combination Ameliorates Lung Emphysema in Experimental Copd Mice

First, it was observed whether the oral administration of LAB containing GMNL-89 and GMNL-133 could ameliorate the LPS/elastase induced pulmonary emphysema. Mice were intranasally challenged with LPS/elastase to induce emphysema. The emphysema was characterized by alveolar wall destruction and abnormally large spaces surrounding the terminal bronchioles (Fig. 1B, COPD group). In the COPD group receiving LAB (Fig. 1B, COPD + LAB), the pulmonary emphysema spaces were restored. The LAB-only group exhibited normal characteristics in lung sections (Fig. 1B, LAB). The MLI of the lung, used to evaluate experimental emphysema [31], was apparently increased in the COPD group (Fig. 1C) but was restored in the COPD + LAB group (Fig. 1C). Thus, our results suggest that the oral administration of *L. reuteri* GMNL-89 and *L. paracasei* GMNL-133 holds potential therapeutic value in the treatment of pulmonary emphysema.

### Oral Administration of a Combination of *L. reuteri *GMNL-89 and *L. paracasei* GMNL-133 Decreases Pulmonary Inflammation in Experimental COPD Mice

**Fig. 2:**
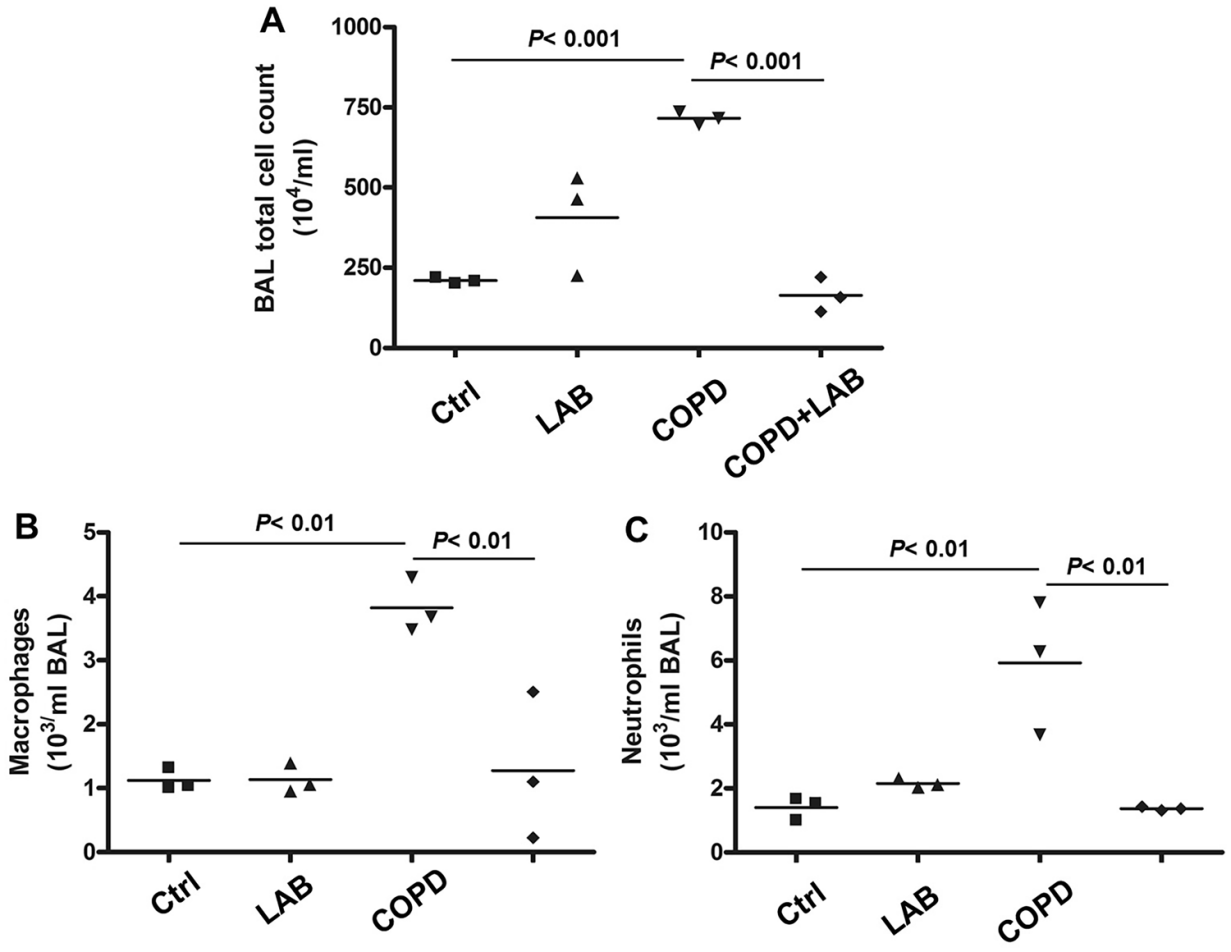
Oral administration of LAB containing GMNL-89 and GMNL-133 reduces macrophage and neutrophil infiltration. BALFs were collected from mice in each group (n = 3 per group), and total cell counts were determined by Giemsa staining (**A**). Infiltrating macrophages (**B**) and neutrophils (**C**) were determined by flow cytometric analysis of CD45 + CD11b + and CD45 + Ly6G + cells, respectively. Ctrl, naïve mice used as control; COPD, intranasal treatment with elastase and LPS; LAB, administration of LAB without induction of COPD; COPD + LAB, induction of COPD followed by oral administration of LAB

**Fig.3:**
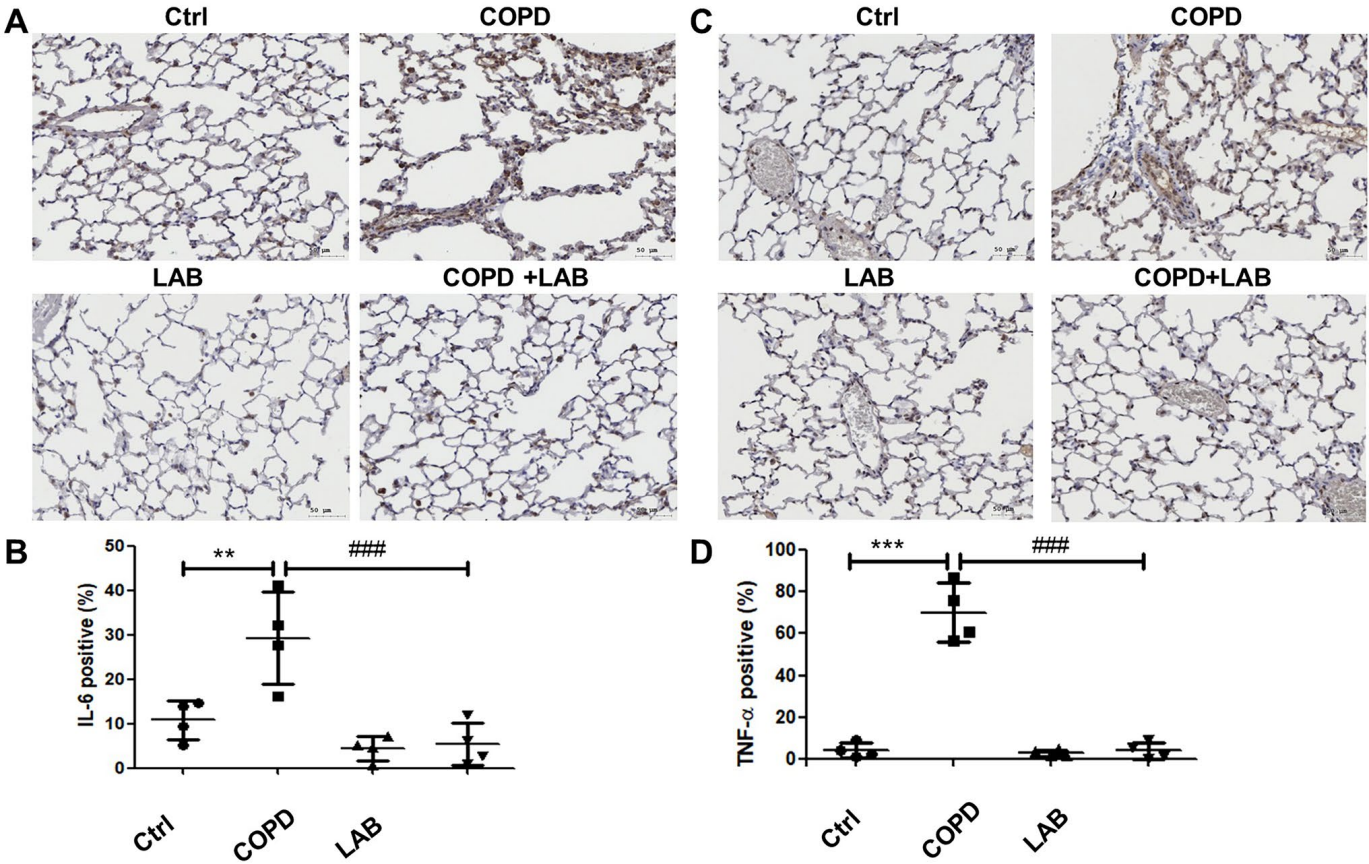
Oral feeding of LAB combination reduces the levels of inflammatory cytokines in lung tissues. The expressions of IL-6 and TNF-α in elastase-LPS-induced COPD mice (n = 4 per group) were determined by immunohistochemistry. (**A**, **C**) The images represent IL-6 (**A**) and TNF-α (**C**) for each group. (**B**, **D**) The quantification results of IL-6 (**B**) and TNF-α (**D**) were analyzed using TissueFAX software. Ctrl, naïve mice used as control; COPD, intranasal treatment with elastase and LPS; LAB, administration of LAB without induction of COPD; COPD + LAB, induction of COPD followed by oral administration of LAB. ** p < 0.01 and *** p < 0.001, compared to Ctrl group. ### *p* < 0.001, compared to COPD group

In addition to emphysema changes, the infiltrations of macrophages and neutrophils in the lung were examined by collecting BALF followed by flow cytometric analysis. The results showed that oral administration of GMNL-89 combined with GMNL-133 significantly reduced total leukocyte infiltration (Fig. 2A) and the levels of infiltrating macrophages (Fig. 2B) and neutrophils (Fig. 2C). IL-6 and TNF-α, known to be important inflammatory mediators in COPD, were observed to be significantly increased in the lung tissue of COPD mice compared to control mice (Fig. 3A). Both the levels of IL-6 (Fig. 3A and B) and TNF-α (Fig. 3C and 3D) were significantly reduced when COPD mice were treated with LAB. Furthermore, the decreased levels of IL-1β, IL-6, and TNF-α in the lung tissues of COPD mice treated with LAB were confirmed by Western blot analysis (Fig. S1). Therefore, the combination of *L. reuteri* GMNL-89 and *L. paracasei* GMNL-133 not only mitigates pulmonary emphysema but also significantly reduces pulmonary inflammation by decreasing leukocyte infiltration and inflammatory cytokine levels in experimental COPD mice, underscoring its therapeutic efficacy.

### *L. reuteri *GMNL-89 and *L. paracasei *GMNL-133 Combination Modulates Gut and Lung Beneficial Bacteria Abundance in Experimental COPD Mice

**Fig. 4:**
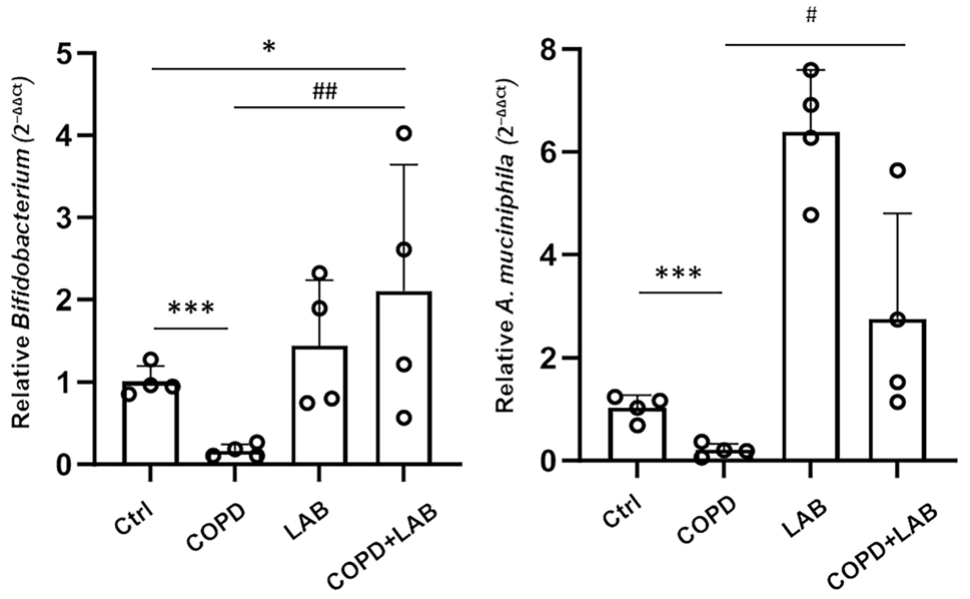
Stool levels of *Bifidobacterium *and *A. muciniphila* were increased in experimental COPD mice after consumption of the LAB combination. Mouse stool DNA samples (n = 4 per group) were collected on the last day after a two-week period of probiotics consumption. The relative levels of *Bifidobacterium* and *A. muciniphila* in stools were determined by qPCR, normalized against total bacteria. Individual data points for each measurement are shown on the graph. Ctrl, naïve mice used as control; COPD, intranasal treatment with elastase and LPS; LAB, administration of LAB without induction of COPD; COPD + LAB, induction of COPD followed by oral administration of LAB. * p < 0.05 compared with Ctrl group. # p < 0.05 compared with the COPD group

Then, the relative abundance of beneficial gut bacteria, *Bifidobacterium* and *A. muciniphila*, was determined by qPCR from stool samples collected on the last day after a two-week period of probiotics consumption. Both *Bifidobacterium* and *A. muciniphila* levels decreased in the stool of COPD mice; however, their levels were restored when the COPD mice were administered LABs (Fig. 4). In the intestine and lung tissues of mice in the four groups, the expression levels of *Lactobacillus* spp., *L. reuteri*, and *L. paracasei* were also determined at this final timepoint. In the intestine, the relative abundance of *L. reuteri* significantly increased in the LAB-only and COPD + LAB groups (Fig. S2A). The abundance of *L. paracasei* also showed an increasing trend following LAB administration, although no statistical difference was observed among the four groups (Fig. S2A). Total *Lactobacillus* spp. abundances did not show significant differences among the groups (Fig. S2A). In the lungs, *L. paracasei* abundance was significantly reduced in COPD mice (Fig. S2B). Total *Lactobacillus* spp. levels were noticeably decreased in COPD mice but were restored in the COPD + LAB group (Fig. S2B).

### Changes in Gut and Lung Microbiota in Experimental COPD Mice After Oral Consumption of *L. reuteri *GMNL-89 and *L. paracasei* GMNL-133 Combination

**Fig. 5:**
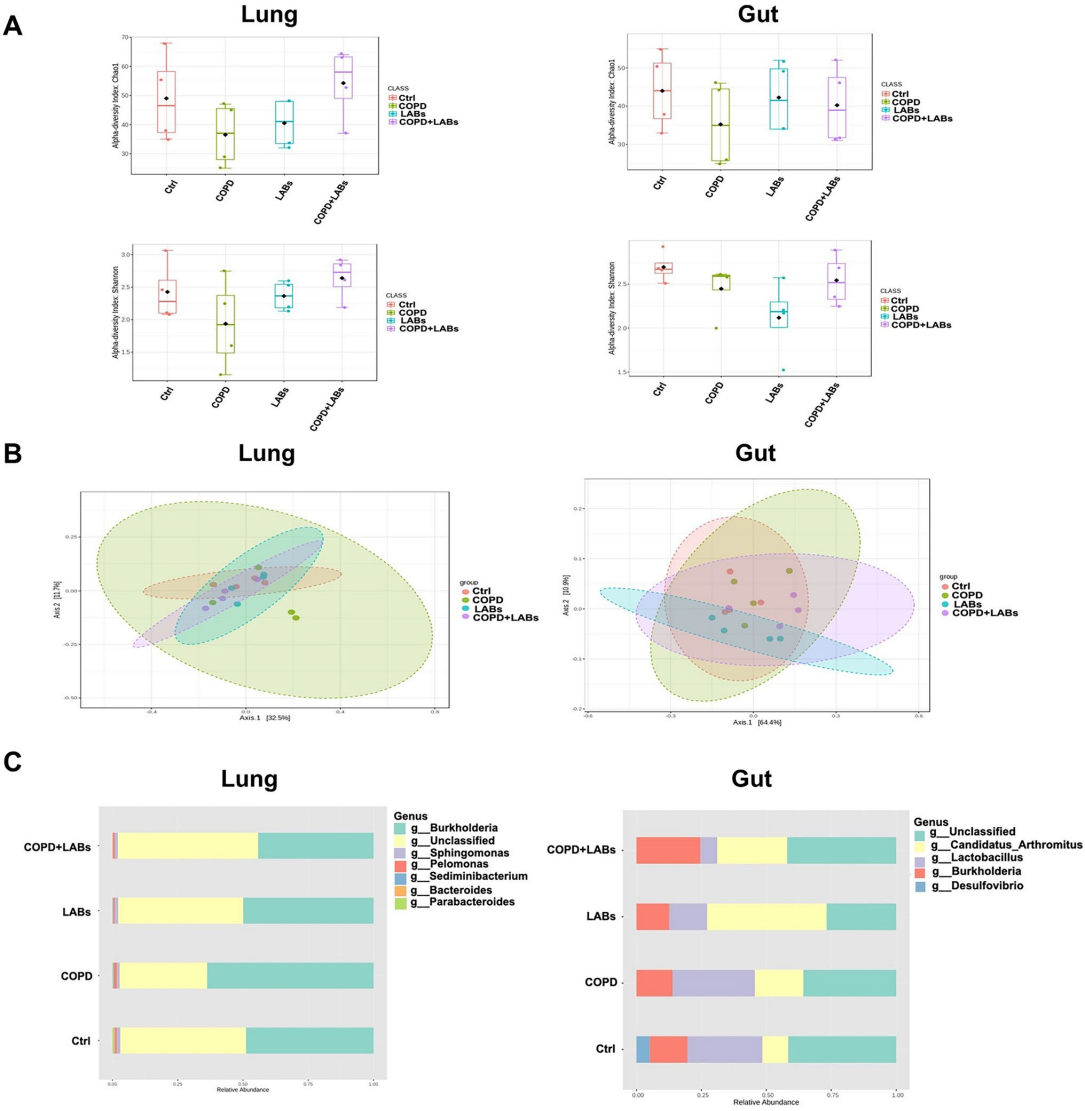
Changes in the gut and lung microbiome of experimental COPD mice after oral consumption of LAB combination. DNA was extracted from mouse lung and intestinal samples (n = 4 per group), and microbiome analysis was performed by NGS of 16S rRNA genes. (**A**) α-diversity was indicated by the Chao1 and Shannon indices. (**B**) β-diversity was measured by PCoA analysis based on weighted UniFrac. (C) The relative abundance at the genus level in the lung and intestine of mice was analyzed by LEfSe. Ctrl, naïve mice used as control; COPD, intranasal treatment with elastase and LPS; LAB, administration of LAB without induction of COPD; COPD + LAB, induction of COPD followed by oral administration of LAB

NGS 16S rRNA gene analysis was used to determine the changes in gut and lung microbiota in experimental COPD mice, with and without the administration of a LAB combination. The alpha diversity of lung and gut microbiota decreased in COPD mice, and this loss of alpha diversity was reversed after consuming the LAB combination (Fig. 5A). Beta diversity results showed no statistical difference among the four groups in lung and gut microbiota (Fig. 5B). We further analyzed the relative abundance of bacterial expression in the four groups by LEfSe. In the lung microbiota, there was an increase in the abundance of the *Burkholderia* genus in the COPD groups compared to the control group. The increase in the abundance of *Burkholderia* was inhibited by the consumption of the LAB combination (Fig. 4C, left panel). In the gut microbiota, *Candidatus arthromitus* increased in COPD mice after consuming LAB compared to the COPD group (Fig. 4C, right). The increase in *C. arthromitus* levels was also observed in the LAB-only group compared to the control group (Fig. 4C, right). Further analysis by LEfSe revealed that the genus *Prevotella* and the family *Lachnospiraceae* had the highest abundance in COPD mice after oral administration of LAB compared to the control mice. In addition, the relative abundance of *Prevotella* genus and *Lachnospiraceae* family was statistically different between the COPD and COPD + LAB groups (Fig. S3).

### Oral Administration of *L. reuteri *GMNL-89 and *L. paracasei* GMNL-133 Combination Affects the Level of SCFAs in the Intestine of Experimental COPD Mice

**Fig. 6:**
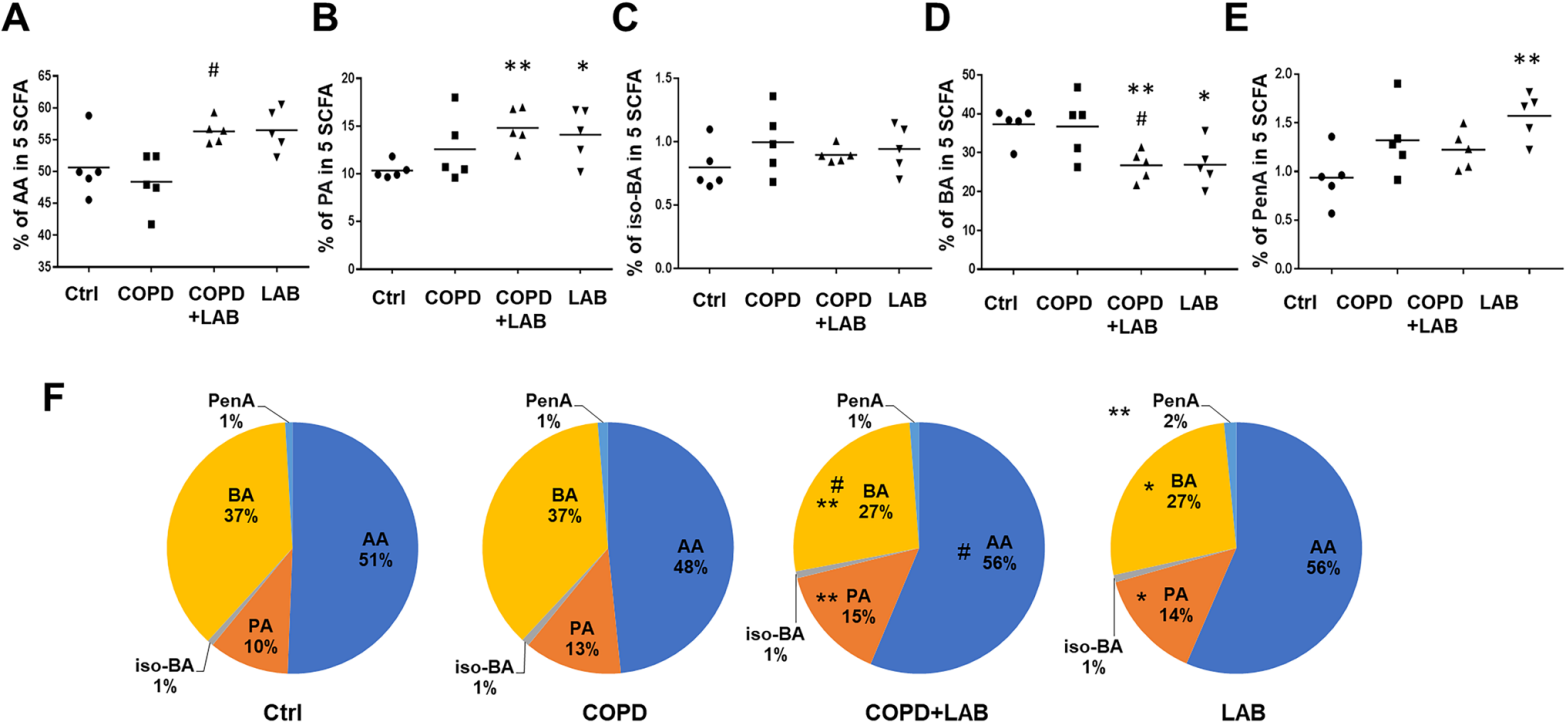
Oral administration of LAB combination significantly increases the SCFA levels of acetic acid and propionic acid in the cecum of experimental COPD mice. Cecal contents (n = 5 per group) were collected to determine SCFAs using the GC-FID method, including acetic acid (AA) (**A**), propionic acid (PA) (**B**), iso-butyric acid (iso-BA) (**C**), butyric acid (BA) (**D**), and pentanoic acid (PenA) (**E**). The pie chart (**F**) shows the percentage of each SCFA. Ctrl, naïve mice used as control; COPD, intranasal treatment with elastase and LPS; LAB, administration of LAB without induction of COPD; COPD + LAB, induction of COPD followed by oral administration of LAB. * p < 0.05 compared with Ctrl group. ** p < 0.01 compared with Ctrl group. # p < 0.05 compared with COPD group

SCFAs are known as important mediators in the gut-lung axis with immunomodulatory effects [32]. The levels of SCFAs, such as AA, PA, iso-BA, BA, and PA, in the cecal samples were detected using the GC-FID method. The individual level of each SCFA relative to the total level of the five SCFAs was measured and presented as a percentage (Fig. 6A to E). The proportions of the five SCFAs among the four groups are presented in a pie chart (Fig. 6F). The percentage of AA was significantly increased in the COPD mice group after consuming LAB, compared to the COPD group (Fig. 6A and F). PA (Fig. 6B) and PenA (Fig. 6E) also increased significantly in the LAB-administered groups, regardless of COPD induction. However, compared with the control mice, the percentage of BA decreased in the two groups receiving LAB (Fig. 6D and F). Among the four groups, there was no statistical difference in the level of iso-BA (Fig. 6C and F).

### Correlations Between Lung Destruction and the Abundance of Bacteria in the Gut and Lungs

**Fig. 7:**
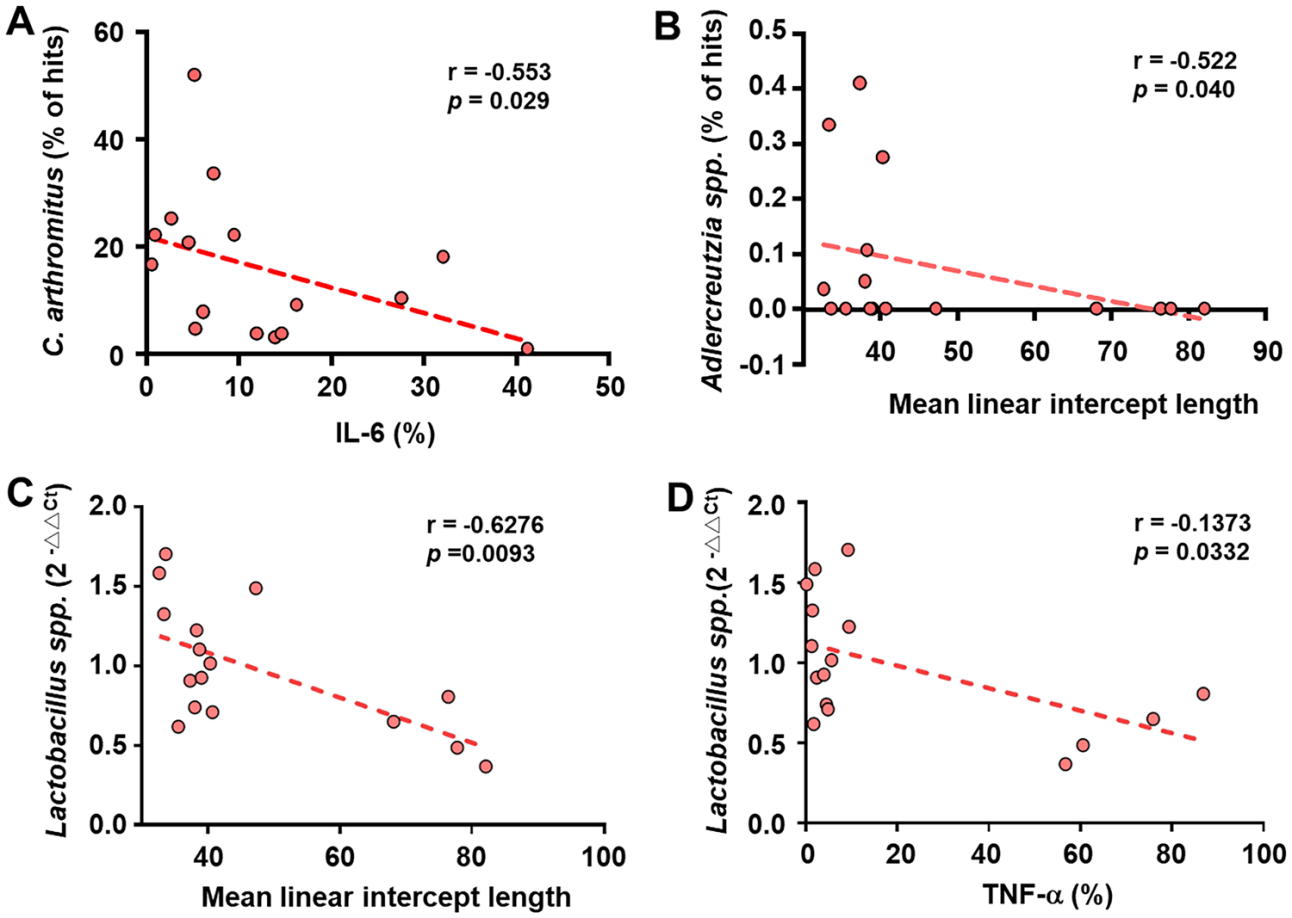
Correlation of emphysema and pro-inflammatory cytokines with beneficial bacteria in the gut or lungs. DNA was extracted from fecal samples (**A**, **B**) and lung tissue (**C**, **D**) (n = 4 per group, total of 16 mice) and was used to detect beneficial bacteria by NGS of 16S rRNA genes (**A**, **B**) or by qPCR with specific primers (**C**, **D**). The levels of IL-6 (**A**) and TNF-α (**D**) in lung tissues were determined by IHC. The levels of pulmonary emphysema were evaluated by mean linear intercept (MLI) length (**B**, **C**). The correlations between *C. arthromitus* and IL-6 (**A**), *Adlercreutzia* and MLI length (**B**), *Lactobacillus* spp. and MLI length (**C**), and *Lactobacillus* spp. and TNF-α (**D**) were determined by Pearson correlation coefficient (r) using SPSS software

Furthermore, we were interested in the correlation between lung inflammation and beneficial bacteria in the gut and lung. Results showed that the inflammatory cytokine IL-6 in the lung was negatively correlated with the abundance of *C. arthromitus* in the gut (Fig. 7A). The estimation of lung emphysema by MLI values displayed a negative correlation with the abundance of *Adlercreutzia* in the gut (Fig. 7B). Additionally, both the MLI values (Fig. 7C) and the inflammatory cytokine TNF-α (Fig. 7D) were negatively correlated with the abundance of *Lactobacillus* spp. in the lung.

## Discussion

### Gut Microbiota and Lung Health Through Gut-lung Axis

In COPD, disrupting the gut-lung axis can lead to dysbiosis of both the gut and lung microbiota, including decreased microbiota diversity, altered microbial composition, increased pro-inflammatory cytokines, and a dysfunctional immune response [33]. In our study, an increased abundance of the phylum Firmicutes and the genera *Lactobacillus* and *C. arthromitus* was observed in the intestines of mice with combined LAB administration (Fig. 5). Gut microbiota may influence the immune response and further regulate airway homeostasis through the gut-lung axis. Recently, a gut microbiome-activated Th17 response against fungi in the respiratory tract was discovered [34, 35]. Interestingly, our results also showed that the lung inflammatory cytokine IL-6 was negatively correlated with the intestinal abundance of *C. arthromitus* (Fig. 7A). *C. arthromitus*, also known as segmented filamentous bacteria, could activate the Th17 response through the release of endocytic vesicles and the transfer of cell wall proteins into the intestine [36, 37]. In addition, we found that the level of pulmonary emphysema was negatively correlated with the abundance of *Adlercreutzia* in the intestine (Fig. 7B). Studies have reported that an increasing abundance of *Adlercreutzia* in the intestine has a beneficial effect on various diseases, including ulcerative colitis, influenza virus infection, and depression [38–40]. Interestingly, *B. animalis* subsp. lactis intervention could increase *Adlercreutzia* abundance, leading to increased acetate and butyrate release into circulation, which in turn increases acetate and butyrate receptor expression and leads to inhibition of lung inflammation and immune cell infiltration [41]. Furthermore, in patients with primary sclerosing cholangitis without concomitant inflammatory bowel disease, a significant decrease in *A. equolifaciens* was observed in the gut microbiota [42]. In our study, a decrease in *Oscillospira* abundance in the intestines was also observed in the COPD group, and this decrease was reversed in COPD mice with LAB administration (data not shown). *Oscillospira*, capable of producing short-chain fatty acids such as butyrate, has recently been suggested as a candidate for next-generation probiotics [43]. The administration of other probiotics, such as *Bacillus coagulans* and *B. subtilis*, has also been shown to positively regulate the abundance of *Oscillospira* in the gut [44, 45]*.* Intestinal *Oscillospira* may regulate the distal organs through circulating route, such as the gut-brain axis, however, the role of *Oscillospira* needs further clarification [46].

### The Pathological Role of Gut *Prevotella* in the Lung Inflammation

In the LEfSe analysis, we found that the genus *Prevotella* and the family *Lachnospiraceae* in the gut had significant influence scores when comparing the COPD and LAB-consuming groups (Fig. S3B). Intestinal *Prevotella* spp. abundance was also decreased in LAB-supplemented COPD mice compared with the COPD group (data not shown). Recently, intestinal *Prevotella* has been associated with chronic inflammatory states [47]. The gut microbiota of smokers contained more *Prevotella* compared to that of non-smokers [48]. Intestinal *Prevotellaceae* also served as colitogenic bacteria that could trigger Th17-mediated inflammation and neutrophil activation in some inflammatory diseases such as periodontitis, rheumatoid arthritis, bacterial vaginosis, intestinal dysbiosis [49]. *Prevotella* might be associated with a presumed COPD subtype linked to the rate of lung function deterioration. By analyzing the gut microbiota from stool samples of COPD patients, the group with declining lung function had a lower proportion of *Prevotella*_9 compared to controls. Moreover, *Prevotella*_2 abundance was further reduced in the decline group after one year [50]. The regulated mechanisms of the intestinal microbiota involved in the decline of lung function needs further investigation.

### The Lung Microbiota in the COPD

The balance of the lung microbiota is important for the maintenance of lung health. Dysbiosis of the lung microbiome has been implicated in the development of lung diseases such as asthma, COPD, lung cancer, and respiratory tract infections [51]. It has been reported that COPD patients have increased levels of *Proteobacteria* and decreased levels of *Firmicutes*, *Bacteroidetes*, *Streptococcus*, *Haemophilus influenzae*, and *Prevotella* spp. compared to healthy subjects [52]. In our study, a correlation between pulmonary emphysema, inflammation, and the expression of lung bacteria was also observed. Interestingly, the abundance of *Lactobacillus* spp. in the lung was negatively correlated with both lung emphysema and the level of the inflammatory cytokine TNF-α in the lung (Fig. 7). This suggests that the *Lactobacilli* in the lungs may play an important role in maintaining lung health. Some in vivo studies have shown that probiotics administered intranasally to the respiratory tract can alleviate lung diseases, indicating that lung *Lactobacillus* is associated with lung microbiome balance [53, 54]. Additionally, intranasal inoculation of lactobacilli in the respiratory tract restored the level of tryptindole-3-acetic acid from tryptophan metabolism, which alleviated the neutrophil-predominant COPD [55].

### SCFA and Lung Function in COPD

SCFAs produced by the gut microbiome play a role in the gut-lung axis by transferring to the lungs and managing lung diseases [56, 57]. For instance, butyrate has broad effects on immune cells in lung disorders such as allergic asthma, COPD, and pulmonary fibrosis [58, 59]. Antunes et al. demonstrated that administering acetate to the respiratory tract could enhance antiviral immunity and had an interferon-enhancing effect to ameliorate the virus-induced inflammatory response during rhinovirus infection [60]. In our study, we analyzed five selected SCFAs in the cecal contents using GC-FID and quantified the concentration of each SCFA based on standards. Consequently, the total SCFA concentration could only be presented as the sum of these five SCFAs. The results indicated that the induction of COPD decreased the total SCFA concentration in the cecal contents, although not to a statistically significant extent. Additionally, administration of LAB did not increase the total SCFA concentration (Fig. S4). Therefore, we focused on analyzing the percentage of each SCFA. Significantly increased proportions of AA were observed after consuming LAB, regardless of whether COPD was induced (Fig. 6A). Furthermore, the proportions of PA and PenA also increased in the LAB-treated group compared to the control mice (Fig. 6B and E), while BA decreased in the LAB-treated group (Fig. 6D). It is known that different gut microbes are responsible for producing different SCFAs. For example, *Firmicutes* species, including *Lactobacillaceae*, *Ruminococcaceae*, and *Lachnospiraceae*, are the primary producers of BA and PA [61, 62], whereas *Bifidobacteria* can metabolize lactulose to produce AA [63]. These altered proportions of individual SCFAs indicate changes in gut microbiota composition following the induction of COPD and oral feeding of LAB. Further studies are needed to determine the role of butyrate in the intranasal elastase/LPS-induced COPD model.

## Conclusion

In this study, the oral administration of the probiotic composition containing *L. reuteri* GMNL-89 and *L. paracasei* GMNL-133 improved pulmonary emphysema and inflammation in mice with LPS/elastase-induced COPD. The diversity of the microbial community in the intestine, altered by the probiotic intervention, could change the content of SCFAs in the intestine. This change may further regulate the lung microbiota and significantly improve lung dysfunction and inflammation via the gut-lung axis. The composition of the probiotics, containing GMNL-89 and GMNL-133, offers an alternative therapeutic avenue for COPD management.

## Supplementary Information

Below is the link to the electronic supplementary material.

Supplementary file1 (PDF 516 KB)

## Data Availability

Data will be made available on request.
