# Supplementary material for: A Lactobacillus Combination Ameliorates Lung Inflammation in an Elastase/LPS—induced Mouse Model of Chronic Obstructive Pulmonary Disease
Source: Probiotics Antimicrob Proteins. 2024 Jun 12;17(5):3416–28. doi: 10.1007/s12602-024-10300-9 (PMC12532766; doi:10.1007/s12602-024-10300-9)
Supplement: Supplementary file 1 — Supplementary file1 (PDF 516 KB) [file 12602_2024_10300_MOESM1_ESM.pdf]

Supplementary information

I. Supporting methods

1. Immunoblotting

Mouse lung tissues were harvested. Tissue extracts were homogenized in tissue protein extraction solution containing 1% proteinase inhibitor cocktail and phosphatase inhibitor cocktail. Protein samples were then analyzed by SDS-PAGE and transferred to polyvinylidene fluoride membranes followed by blocking with PBS containing 0.1% Tween-20 (PBS-T) solution with 5% skim milk (w/v) for 1 h at room temperature. After washing with PBS-T, membranes were incubated overnight with primary antibodies of IL-1β, IL-6, and TNF-α, respectively. After washing steps, the membranes were incubated with horseradish peroxidase-conjugated secondary antibodies for 1 hour at room temperature, followed by development of chemoluminescence signals with ECL substrate and imaging with a digital imaging system (FUSION SOLO S, Vilber, Marne-la-Vallée cedex 3, France).

II. Supporting data

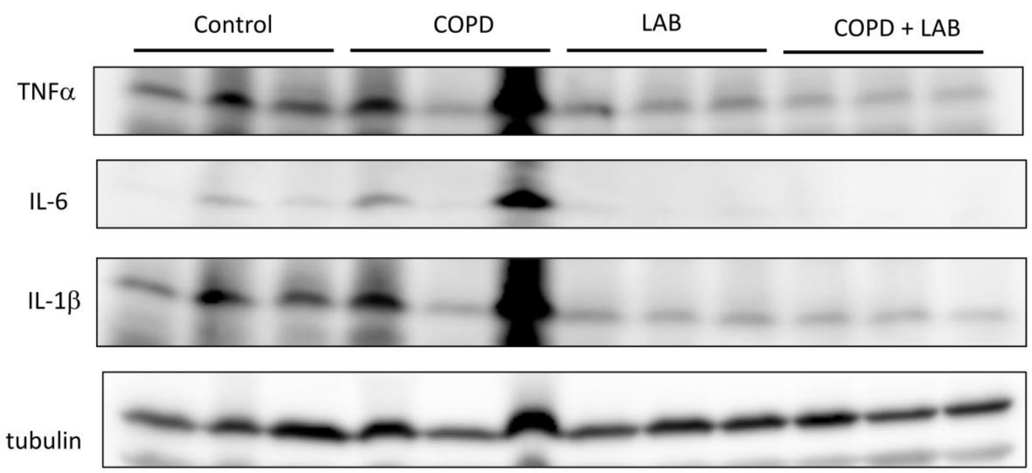

Fig. S1. Reduction of TNF-α/IL-1β/IL-6 expression in lung tissue of LPS/elastase-induced COPD mice by oral LAB combination feeding. 30 μg total protein extracts from lung tissues were used for Western blot analysis of the expression of the indicated cytokines. Tubulin was used as a protein loading control. Ctrl, naïve mice used as control; COPD, intranasal treatment with elastase and LPS; LAB, administration of LAB without induction of COPD; COPD+LAB, induction of COPD followed by oral administration of LAB.

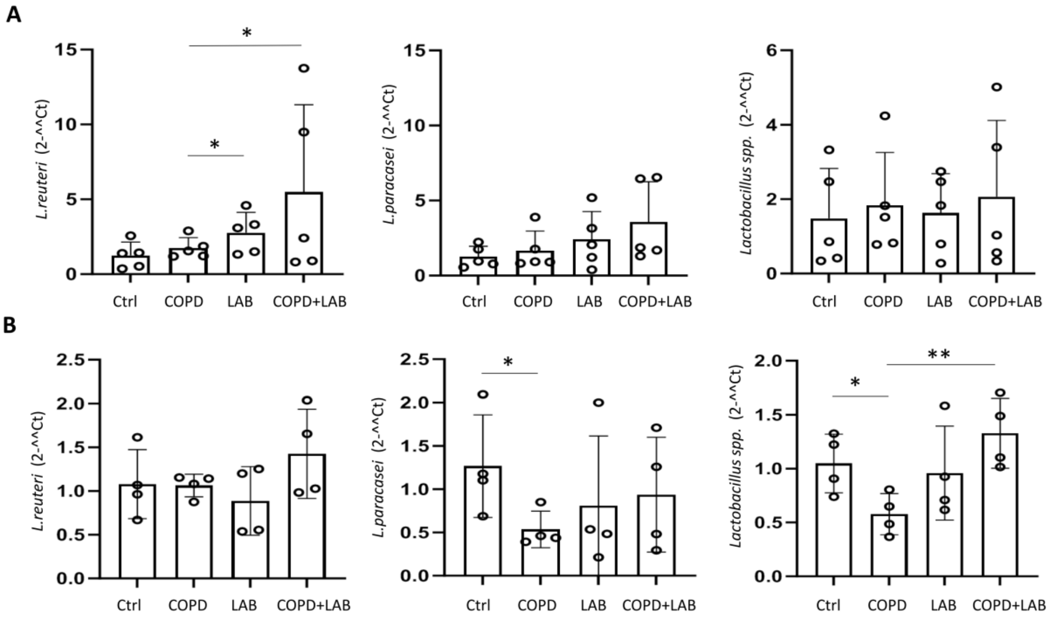

Fig. S2. Relative levels of *L. reuteri*, *L. paracasei*, and *Lactobacillus* spp. in mouse intestine and lung tissues. Total *Lactobacillus* spp., *L. reuteri*, or *L. paracasei* levels after 2 weeks of probiotics consumption were quantified by qPCR using DNA extracted from intestinal samples of 5 mice (A) or lung tissues of 4 mice (B). Individual data points for each measurement are shown on the graph. Ctrl, naïve mice used as control; COPD, intranasal treatment with elastase and LPS; LAB, administration of LAB without induction of COPD; COPD+LAB, induction of COPD followed by oral administration of LAB. *, $p < 0.05$; **, $p < 0.01$ compared with the Ctrl group.

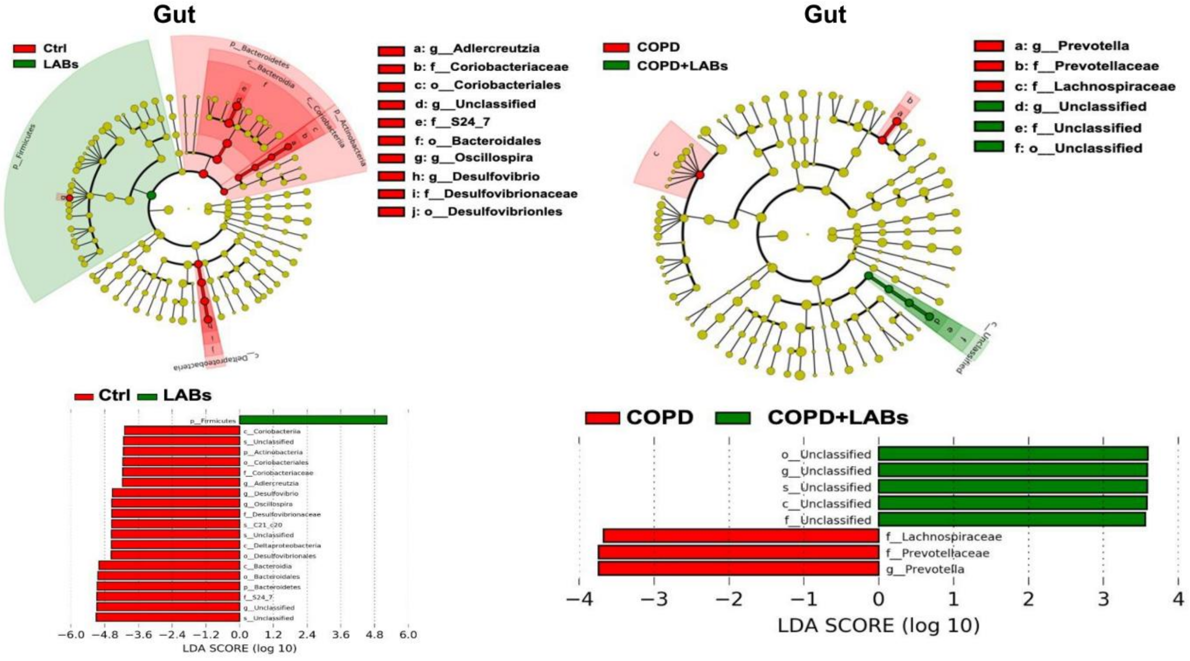

Fig. S3. Significant differences between COPD and COPD with oral LAB combination were observed in the gut genera *Prevotella* and *Lachnospiraceae*. The cladograms showed the phylogenetic relationships of the bacterial taxa as revealed by LEfSe. The bar chart presented the log-transformed LDA scores of the bacterial taxa identified by the LEfSe analysis with a cut-off value of 2.0 by means of Speraman's correlation. Ctrl, naïve mice used as control; COPD, intranasal treatment with elastase and LPS; LABs, administration of LAB without induction of COPD; COPD+LABs, induction of COPD followed by oral administration of LAB.

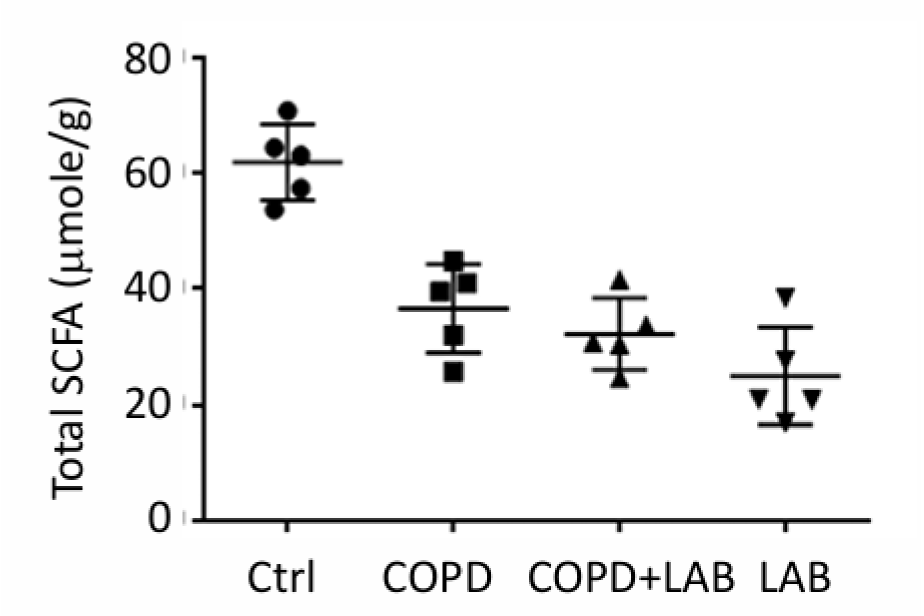

Figure S4. Total SCFA level was not increased after oral consumption of LAB. To obtain the total SCFA levels for each experimental group, the concentrations of five SCFAs in Figure 6 were summed. Ctrl, naïve mice used as control; COPD, intranasal treatment with elastase and LPS; LAB, administration of LAB without induction of COPD; COPD+LAB, induction of COPD followed by oral administration of LAB. There was no statistical difference between the ctrl group and the COPD group, nor between the COPD group and the COPD+LAB group, when calculated by one-way ANOVA with Kruskal-Wallis test.
